# 伴ETV6::ABL1融合的髓系/淋系肿瘤1例报告并文献复习

**DOI:** 10.3760/cma.j.cn121090-20250122-00040

**Published:** 2025-07

**Authors:** 文文 李, 扬 刘, 倩 江

**Affiliations:** 1 北京大学人民医院青岛医院血液中心、青岛大学附属妇女儿童医院，青岛 266000 Department of Hematlogy, Peking University People's Hospital Qingdao Hospital, Women and Children's Hospital, Qingdao University, Qingdao 266000, China; 2 北京大学人民医院血液科,北京大学血液病研究所，国家血液系统疾病临床医学研究中心，北京 100044 Department of Hematology, Peking University People's Hospital, Peking University Institute of Hematology, National Clinical Center of Hematologic Disease, Beijing 100044, China

## Abstract

为加深对伴ETV6::ABL1融合的髓系/淋系肿瘤的认识，回顾性分析1例伴ETV6::ABL1融合的髓系/淋系肿瘤患者的病例资料，并对相关文献进行复习。患者，男，33岁，因“乏力10个月”就诊，完善检查，诊为“不典型慢性髓性白血病”。给予伊马替尼400 mg每日1次口服治疗。根据WHO第五版造血淋巴肿瘤分类和2022年国际共识分类，调整诊断为“伴ETV6::ABL1融合的髓系/淋系肿瘤”。经治疗，患者乏力症状明显改善，ETV6::ABL1融合基因转阴。伴ETV6::ABL1融合的髓系/淋系肿瘤极其罕见，临床类似于慢性髓系白血病，容易误诊、漏诊。

## Abstract (en)

To enhance understanding of myeloid/lymphoid neoplasms with ETV6::ABL1 fusion, we retrospectively analyzed the clinical data of a patient with myeloid lymphoid neoplasms with ETV6::ABL1 fusion. A review of the relevant literature was also conducted. The patient, a 33-year-old male, presented with a “10-month history of fatigue” and was initially diagnosed with “atypical chronic myeloid leukemia” following comprehensive clinical evaluation. Treatment with imatinib（400 mg once daily）was initiated. Based on findings from the literature and in accordance with the “World Health Organization 5th edition classification of hematopoietic and lymphoid tumors” and “the 2022 International Consensus Classification,” the diagnosis was revised to “myeloid/lymphoid neoplasm with ETV6::ABL1 fusion（M/LN-ETV6::ABL1）.” Following treatment, the patient's fatigue significantly improved, and the fusion gene became undetectable. M/LN-ETV6::ABL1 fusion are extremely rare and often clinically resemble CML, making them susceptible to misdiagnosis and underdiagnosis.

ETV6::ABL1融合基因是一种罕见的重现性遗传学异常，存在于儿童和成人的部分血液系统恶性肿瘤中。t（9；12）（q34；p13），即位于9q34的ABL1与12p13的ETV6融合后可以持续性激活酪氨酸激酶ABL1、促进细胞增殖和转化。ETV6::ABL1融合基因可发生于急性白血病、髓系/淋系肿瘤伴嗜酸细胞增多[1]–[2]。WHO第五版造血淋巴肿瘤分类和2022年国际共识分类新增疾病亚类“伴ETV6::ABL1融合的髓系/淋系肿瘤”。本病罕见，患者早期无特异性临床表现，易漏诊、误诊。现报道1例患者的诊疗经过并进行文献复习，以提高对此类疾病的认识。

## 病例资料

患者，男，33岁，因“乏力10个月”于2023年2月就诊于我院血液科。患者2022年4月无诱因出现乏力、纳差、盗汗，半年体重下降10 kg，2022年10月于当地某三甲医院检查血常规：WBC 122.71×10^9^/L，HGB 83 g/L，PLT 159×10^9^/L，嗜酸性粒细胞比例1.9％，嗜酸性粒细胞绝对值2.32×10^9^/L。血涂片嗜酸性粒细胞未见。乳酸脱氢酶696 U/L。骨髓象：增生极度活跃，原始细胞占1.5％，粒系占94.5％，各阶段可见，嗜酸性粒细胞明显增多，嗜酸性中幼、晚幼、杆状粒细胞分别占6.5％、3.0％、9.0％，嗜碱性粒细胞占1.5％，单核细胞占2.5％，不除外慢性髓性白血病（CML）。骨髓免疫分型可见中性粒细胞比例增高达80.7％，嗜酸、嗜碱性粒细胞比例增高；染色体示46，XY，del（6）（q21q23）[5]/46，XY[1]，BCR::ABL1（p190、p210、p230）及JAK2p.V617F、JAK2 exon12、CALR、MPL均阴性。血液肿瘤多种融合基因检查示ETV6::ABL1定性阳性。查体：浅表淋巴结、肝未触及，脾肋缘下9 cm。诊为“不典型CML”。2022年10月给予伊马替尼400 mg每日1次口服。患者2023年2月就诊于我院血液科，复查血常规：WBC 6.71×10^9^/L，HGB 152 g/L，PLT 123×10^9^/L，血涂片可见嗜酸性粒细胞占1％。骨髓穿刺活检显示造血组织容量50％，红系、粒系增生活跃，MF-0级。骨髓涂片：增生低下，原始粒细胞以下细胞可见；ETV6::ABL1定量（骨髓）14.7％。髓系肿瘤二代测序：ZRSR2突变，变异丰度2.6％。染色体核型分析：46，XY，del（6）（q21q25）[3] / 46，XY[17]。综合患者病史及特点诊断“伴ETV6::ABL1融合的髓系/淋系肿瘤”，继续予伊马替尼400 mg每日1次口服。2023年4月复查，染色体核型分析（骨髓）：46，XY[20]，ETV6::ABL1定量（骨髓）2.5％。2023年11月ETV6::ABL1定量（外周血）0。2024年3月血常规：WBC 5.25×10^9^/L，HGB 158 g/L，PLT 125×10^9^/L。ETV6::ABL1定量（外周血）0。目前继续伊马替尼400 mg每日1次口服，无不适。

## 讨论及文献复习

ETV6::ABL1可见于成人及儿童急性髓系、淋系白血病，也可见于骨髓增殖性肿瘤。第五版WHO分类及国际共识分类新增伴ETV6::ABL1、JAK2重排、FLT3重排及其他酪氨酸激酶融合的髓系/淋系肿瘤。由于既往并无单独的共识分类，故常诊断为不典型CML伴ETV6::ABL1、骨髓增殖性肿瘤不能分类伴ETV6::ABL1、骨髓增生异常综合征/骨髓增殖性肿瘤伴ETV6::ABL1。本例患者具有骨髓增殖性疾病的特点、不符合经典的CML、骨髓纤维化、慢性粒-单核细胞白血病、慢性中性粒细胞白血病等的诊断，具有ETV6::ABL1融合基因，符合第五版WHO分类及国际共识分类新亚类“伴ETV6::ABL1融合的髓系/淋系肿瘤”诊断标准。

ETV6基因也称为TEL基因，是位于12p13的转录因子E26转化特异性家族（ETS）的成员。它与40多种不同染色体带有关联，并在白血病的发生发展中起作用[3]。ETV6与ABL1融合具有两种异构体：A型异构体是ETV6外显子4与ABL1外显子2的融合；B型异构体是ETV6外显子5与ABL1外显子2的融合[4]–[6]。因ETV6外显子5包含GRB2的SH2结构域的直接结合位点，从而增强了PI3激酶和MAP激酶途径，具有更高的激酶活性[6]。ETV6通过诱导ETV6::ABL1蛋白分子指向的氨基末端结构域寡聚化，使ABL的酪氨酸激酶活性失调，类似于BCR的二聚化结构域。ETV6::ABL1融合基因活化的信号转导通路部分与BCR::ABL1p210相似[7]。该融合导致酪氨酸激酶结构域的激活[4],[8]。这两种融合都可以启动相似的下游事件，促进细胞增殖及存活[9]–[10]。因此，ETV6::ABL1融合基因和BCR::ABL1融合基因阳性的患者有着相似的临床和细胞形态学特点：核左移的高白细胞、嗜酸性粒细胞增多（80％的患者>1.5×10^9^/L）、脾大、骨髓增生活跃和纤维化[11]。体外实验表明，伊马替尼能抑制ETV6::ABL1融合基因阳性细胞的生长，这进一步证实ETV6::ABL1和BCR::ABL1功能的相似性[12]。

研究表明，伊马替尼通过在细胞内抑制酪氨酸磷酸化，在体外和体内均表现出对ABL酪氨酸激酶的强效选择性抑制作用，具有显著的抗增殖和抗肿瘤活性。伊马替尼对经典的BCR::ABL（p210）体外半抑制浓度（IC_50_）为0.25 µmol/L，而对表达ETV6::ABL1的细胞系体外也具有抑制增殖作用，IC_50_为0.35 µmol/L[13]–[14]。类似的，携带ETV6::ABL1融合基因的细胞对达沙替尼表现出显著的敏感性，IC_50_为0.29 µmol/L[15]。基于以上理论基础，一线选择伊马替尼的患者若疗效不佳或出现疾病进展可调整为达沙替尼或其他酪氨酸激酶抑制剂进行治疗。

本例患者染色体显带并未发现典型t（9；12）（q34；p13），但是可见ETV6::ABL1融合基因。ETV6::ABL1融合基因相对于其天然染色体上的着丝粒处于相反的转录方向，至少需要三次断裂和重新拼接才能形成ABL1到ETV6的框内融合转录本[16]。正是因为这种复杂性、隐匿性，应用常规G显带法很难发现这种细胞遗传学异常，ETV6::ABL1融合的发生率可能被低估，甚至由于隐匿插入，在FISH检测为假阴性[17]。故推荐转录组深度测序或多重PCR法进行检测。本例患者初诊时应用G显带法未发现典型融合，通过PCR法鉴定ETV6::ABL1融合。

我们对伴ETV6::ABL1融合的髓系/淋系肿瘤的中英文文献进行了检索，通过检索PubMed、Cochrane Library、中国知网、万方数据库，截至2025年1月2日已发表中英文文献60余篇，由于历史跨度较长，我们对处于慢性期并一线接受酪氨酸激酶抑制剂治疗的相关报道进行了汇总[18]–[26]。共22例患者，一线接受伊马替尼治疗16例、尼罗替尼2例、达沙替尼4例。对有完整资料的20例患者进行总结分析，中位随访22个月（范围：1个月～10年），5例患者转为急性白血病，中位5年总生存率54.3％，4年无进展生存率48.4％。汇总分析结果显示伴ETV6::ABL1融合的髓系/淋系肿瘤且处于慢性期的患者一线接受酪氨酸激酶抑制剂疗效优于既往接受其他治疗的报道[11]。参考CML，异基因造血干细胞移植在这一疾病中的地位可能需要根据疗效（尤其是ETV6::ABL1定量变化）来确定。

## References

[1] O'Brien SG, Vieira SA, Connors S (2002). Transient response to imatinib mesylate (STI571) in a patient with the ETV6-ABL t(9;12) translocation[J]. Blood.

[2] Yamamoto K, Yakushijin K, Nakamachi Y (2014). Extramedullary T-lymphoid blast crisis of an ETV6/ABL1-positive myeloproliferative neoplasm with t(9;12)(q34;p13) and t(7;14)(p13;q11.2)[J]. Ann Hematol.

[3] Nand R, Bryke C, Kroft SH (2009). Myeloproliferative disorder with eosinophilia and ETV6-ABL gene rearrangement: efficacy of second-generation tyrosine kinase inhibitors[J]. Leuk Res.

[4] Choi SI, Jang MA, Jeong WJ (2017). A Case of Chronic Myeloid Leukemia With Rare Variant ETV6/ABL1 Rearrangement[J]. Ann Lab Med.

[5] Park J, Kim M, Lim J (2013). Variant of ETV6/ABL1 gene is associated with leukemia phenotype[J]. Acta Haematol.

[6] Million RP, Harakawa N, Roumiantsev S (2004). A direct binding site for Grb2 contributes to transformation and leukemogenesis by the Tel-Abl (ETV6-Abl) tyrosine kinase[J]. Mol Cell Biol.

[7] Voss J, Posern G, Hannemann JR (2000). The leukaemic oncoproteins Bcr-Abl and Tel-Abl (ETV6/Abl) have altered substrate preferences and activate similar intracellular signalling pathways[J]. Oncogene.

[8] Kakadia PM, Schmidmaier R, Völkl A (2016). An ETV6-ABL1 fusion in a patient with chronic myeloproliferative neoplasm: Initial response to Imatinib followed by rapid transformation into ALL[J]. Leuk Res Rep.

[9] Voss J, Posern G, Hannemann JR (2000). The leukaemic oncoproteins Bcr-Abl and Tel-Abl (ETV6/Abl) have altered substrate preferences and activate similar intracellular signalling pathways[J]. Oncogene.

[10] Yokota A, Hirai H, Shoji T (2017). Constitutively active ABL family kinases, TEL/ABL and TEL/ARG, harbor distinct leukemogenic activities in vivo[J]. Leukemia.

[11] Schwaab J, Naumann N, Luebke J (2020). Response to tyrosine kinase inhibitors in myeloid neoplasms associated with PCM1-JAK2, BCR-JAK2 and ETV6-ABL1 fusion genes[J]. Am J Hematol.

[12] Carroll M, Ohno-Jones S, Tamura S (1997). CGP 57148, a tyrosine kinase inhibitor, inhibits the growth of cells expressing BCR-ABL, TEL-ABL, and TEL-PDGFR fusion proteins[J]. Blood.

[13] Buchdunger E, Zimmermann J, Mett H (1996). Inhibition of the Abl protein-tyrosine kinase in vitro and in vivo by a 2-phenylaminopyrimidine derivative[J]. Cancer Res.

[14] Druker BJ, Tamura S, Buchdunger E (1996). Effects of a selective inhibitor of the Abl tyrosine kinase on the growth of Bcr-Abl positive cells[J]. Nat Med.

[15] Uemura S, Nishimura N, Hasegawa D (2018). ETV6-ABL1 fusion combined with monosomy 7 in childhood B-precursor acute lymphoblastic leukemia[J]. Int J Hematol.

[16] Van Limbergen H, Beverloo HB, van Drunen E (2001). Molecular cytogenetic and clinical findings in ETV6/ABL1-positive leukemia[J]. Genes Chromosomes Cancer.

[17] Saft L, Kvasnicka HM, Boudova L (2023). Myeloid/lymphoid neoplasms with eosinophilia and tyrosine kinase fusion genes: A workshop report with focus on novel entities and a literature review including paediatric cases[J]. Histopathology.

[18] Gancheva K, Virchis A, Howard-Reeves J (2013). Myeloproliferative neoplasm with ETV6-ABL1 fusion: a case report and literature review[J]. Mol Cytogenet.

[19] Kawamata N, Dashti A, Lu D (2008). Chronic phase of ETV6-ABL1 positive CML responds to imatinib[J]. Genes Chromosomes Cancer.

[20] Kelly JC, Shahbazi N, Scheerle J (2009). Insertion (12;9)(p13;q34q34): a cryptic rearrangement involving ABL1/ETV6 fusion in a patient with Philadelphia-negative chronic myeloid leukemia[J]. Cancer Genet Cytogenet.

[21] De Braekeleer E, Douet-Guilbert N, Rowe D (2011). ABL1 fusion genes in hematological malignancies: a review[J]. Eur J Haematol.

[22] Yao J, Xu L, Aypar U (2021). Myeloid/lymphoid neoplasms with eosinophilia/ basophilia and ETV6-ABL1 fusion: cell-of-origin and response to tyrosine kinase inhibition[J]. Haematologica.

[23] Mori N, Ohwashi-Miyazaki M, Okada M (2016). Translocation (9;12)(q34.1;p13.?3) Resulted in ETV6-ABL1 Fusion in a Patient with Philadelphia Chromosome-Negative Chronic Myelogenous Leukemia[J]. Acta Haematol.

[24] Qi Z, Smith C, Shah NP (2023). Complex Genomic rearrangements involving ETV6::ABL1 gene fusion in an individual with myeloid neoplasm[J]. Genes (Basel).

[25] Chen F (2024). ETV6::ABL1 positive myeloid/lymphoid neoplasms with eosinophilia and tyrosine kinase gene fusions (MLN-TK) with blast crisis treated with flumatinib mesylate[J]. Ann Hematol.

[26] Bochicchio MT, Marconi G, Baldazzi C (2023). ETV6::ABL1-Positive Myeloid Neoplasm: A Case of a Durable Response to Imatinib Mesylate without Additional or Previous Treatment[J]. Int J Mol Sci.

